# The Relationship between Life Stress and Breastfeeding Outcomes among Low-Income Mothers

**DOI:** 10.1155/2012/902487

**Published:** 2012-12-31

**Authors:** Ann M. Dozier, Alice Nelson, Elizabeth Brownell

**Affiliations:** ^1^Department of Public Health Sciences, University of Rochester, 265 Crittenden Boulevard., CU 420644, Rochester, NY 14642-0644, USA; ^2^Department of Research, Connecticut Children's Medical Center, 282 Washington Street, Hartford, CT 06106, USA

## Abstract

Stressful life events during pregnancy negatively affect maternal and infant outcomes including breastfeeding initiation. Their impact on breastfeeding duration is uncertain. Given breastfeeding's important health benefits we analyzed stressful life event types and cessation of any and exclusive breastfeeding by 4 and 13 weeks. *Methods*. We collected self-administered survey data at 5–7 months postpartum from over 700 primarily urban low-income US mothers. Data covered prepregnancy, prenatal, and postpartum periods including 14 stressful life events (categorized into financial, emotional, partner-associated, traumatic). Analyses included only mothers initiating breastfeeding (*n* = 341). Logistic regressions controlled for maternal characteristics including a breastfeeding plan. *Results*. All four stress categories were associated with shorter duration of any and exclusive breastfeeding. In the adjusted models, statistically significant relationships remained for financial stress (4 weeks cessation of any breastfeeding duration) and traumatic stress (13 weeks exclusive breastfeeding cessation). Controlling for stress, *a longer* breastfeeding plan was significantly associated with *a shorter* breastfeeding duration (all models) as was depression during pregnancy and current smoking (several models). *Conclusions*. Among low-income women, impact of stressful life events on cessation of breastfeeding may differ by stress type and interfere with achievement of breastfeeding goal. Among these stressed mothers, breastfeeding may serve as a coping mechanism.

## 1. Introduction

 Optimal breastfeeding duration and exclusivity practices contribute to significant short- and long-term health benefits for both mother and baby [1, 2]. Current professional associations, including the World Health Organization recommend exclusive breastfeeding for 6 months and continued breastfeeding for at least a year [3, 4]. In the USA, efforts by professional, government, and health and human service organizations to increase breastfeeding rates resulted in increasing initiation rates [5, 6]. Duration and exclusivity remain well below national goals, especially among low-income mothers [7]. 

 Numerous factors influence breastfeeding outcomes from institutional practices to individual characteristics and actions [8–10]. The latter include demographic and maternal factors such as maternal race/ethnicity (non-white and/or Hispanic) [11], less education [12], elevated prepregnancy Body Mass Index (BMI) [13], language (English speaking) [14], and younger age [11], all of which are associated with early cessation of any and exclusive breastfeeding in the USA. Other known factors linked with early cessation include: poor sense of breastfeeding self-efficacy [15], smoking [16, 17], alcohol use [18], maternal depression [19, 20], and not having a prenatal breastfeeding plan [18, 21]. Of note, low-income mothers are more likely to experience several of these factors, possibly contributing to their earlier cessation of breastfeeding.

 The extent to which mothers experience stressful life events and how these may be related to breastfeeding outcomes is less well understood. Stressful life events known to be associated with other negative or adverse perinatal outcomes include small for gestational age infants [22], gestational diabetes [23], increased risk of physical abuse [24], low birth weight and preterm birth [25], behavior problems for the infant later in life [26], and maternal depression [27, 28]. Research describing associations between stressful life events and breastfeeding cessation is limited. For example, within the maternal depression and breastfeeding literature, stressful life events are typically only used as an adjusted factor and not a primary exposure [27]. Additionally, most studies are not specific to low-income women.

To our knowledge, only one study examined whether stressful life events during pregnancy influenced breastfeeding rates [29]. Stress in that study was quantified as a total stress score and showed that independent of maternal characteristics more stressful life events were significantly associated with earlier breastfeeding cessation. Given that stress is a complex phenomenon, a different approach may be warranted such as the one developed by Ahluwalia et al. (2001). Their methodology categorized stressful life events into four types (financial, emotional, traumatic, and partner associated) [22]. We chose this methodology instead of using a total life stress score. The latter, by weighting each event the same, assumes that each event is equivalent, which may not be the case. Further we were interested in observing how different types of stressful life events were associated with breastfeeding outcomes.

The purpose of this analysis was to examine the association between four types of stressful life events and breastfeeding outcomes (cessation of any and exclusive breastfeeding) among low-income mothers who initiated breastfeeding.

## 2. Methods

### 2.1. Setting

 Mothers in this study resided in an upstate New York county, with a major metropolitan city and around 8000 births annually, of which approximately 40% occur to low-income mothers. We defined low-income as mothers prenatally enrolled in a food assistance program (Women's, Infants and Children's Supplemental Nutrition Program, WIC) and/or with deliveries paid for by publically funded health insurance programs (i.e., Medicaid). Four urban pediatric and one family medicine practice serving primarily low-income clientele assisted with survey distribution.

### 2.2. Sample

Eligible mothers were at least 5 months postdelivery of a live infant with residence in the county. Other than mothers' name and address no other demographic or contact information was provided by the practices about the mothers or index infants.

### 2.3. Data Collection

Participating practices identified all eligible mothers and provided electronic files with contact names and addresses. Survey distribution occurred through mailings to all mothers with usable addresses. The first mailing occurred between 5 and 7 months postpartum. Followup was restricted to a second mailing at 3 weeks. Telephone numbers were not provided so other types of followup were not possible. To incentivize participation, respondents were eligible to participate in a raffle. 

### 2.4. Survey Instrument

The “You and Your Baby” Survey (YYBS) gathered surveillance data from mothers. In addition to demographics, the YYBS captured prepregnancy, prenatal, and postpartum information, using items primarily from the US Centers for Disease Control's Pregnancy Risk Assessment Monitoring System (version 6) [30] and items used in the primary author's previous surveys. 

Following the first year of distribution, the YYBS included additional questions such as an adapted version of the Wagnild and Young's resiliency scale (16 items) [31, 32] and the “Moods and Feelings During Pregnancy Life Events Self-Evaluation” scale (33 events) [28]. The final survey for year two included 83 questions. Surveys were mailed with a cover letter and stamped self-addressed return envelope.

### 2.5. Stress Categories

The “Moods and Feelings during Pregnancy Life Events Self Evaluation” scale started with the question “Did any of these things happen during your pregnancy or in the first month of your baby's life?” Each of the 33 events listed included three responses: “No,” “Yes, affected me a little,” or “Yes, affected me a lot.” Using a previously published methodology [22], 14 of the 33 events served as the basis to create four stress categories: partner-associated (three items: separated from husband/partner, argued with husband/partner more often, husband/partner said he did not want pregnancy), traumatic (five items: stayed in a shelter or needed emergency shelter, physical fights with partner/husband or someone else, husband/partner had legal trouble and/or went to jail, mother had legal trouble and/or went to jail, someone very close had a problem with drinking or drugs), financial (two items: big problems with money, moved to a new address), and emotional (four items: family member had serious accident/illness, husband/partner had serious accident/illness, husband/partner died, friend/relative died). Respondents were classified as exposed if they responded “Yes” to any questions contained within each stress type.

### 2.6. Breastfeeding Outcomes

Mothers reported if they were currently breastfeeding and if not when they stopped (based on infant age). This information was used to calculate if the mother was still breastfeeding at 4 and 13 weeks (yes/no). Mothers also reported if and when the baby was introduced to formula, other liquids cereal, or solid foods. Using this information, exclusive breastfeeding duration was calculated at 4 and 13 weeks (yes/no) with exclusivity being defined as the infant receiving only breast milk and no other liquid or solid food. 

### 2.7. Analyses

 We conducted an exploratory analysis using bivariate analyses and multivariable logistic regression models utilizing mothers who initiated breastfeeding that completed the modified survey. We first analyzed the crude relationship between each of the 4 types of stress categories and cessation of any or exclusive breastfeeding (at 4 and 13 weeks). Final regression models included the exposure (one of the stress categories) and maternal variables identified through previous breastfeeding research: demographics including maternal age, race, language, education, and inner-city residence (as defined by 9 urban zip codes with elevated levels of poverty, locally referenced to as “The Crescent”) [11, 12, 14], breastfeeding factors (prior experience and prenatal plan) [15, 18, 21], and maternal characteristics (prepregnancy BMI score, self-reported depression during or right after most recent pregnancy, alcohol use, and smoking habits) [13, 16–20]. No sample size calculation was undertaken since this was a secondary analysis of a convenience sample. All analyses were conducted using SPSS version 19 with a 2-sided *P*  value set at <0.05. Approval for these analyses was obtained from the University of Rochester's IRB.

## 3. Results

Over the two years, 21.0% of all eligible mothers responded (*n* = 760), of which 499 completed the modified survey with 341 reporting ever breastfeeding. As depicted in Table 1 these mothers had a mean age of 26.3 and were primarily Black race (39.6%), spoke only English at home (70.6%), had a high school education or more (74.5%), and resided within the inner-city (64.5%). Other maternal factors included having a breastfeeding plan of 4 or more months (55.9%), being primiparous (46.0%), self-reporting no depression during or right after most recent pregnancy (73.6%), having a prepregnancy BMI score of less than 25.0 (46.6%), currently using alcohol (49.9%), and currently smoking (25.1%). Out of the 14 identified stressful life events, the average number of events experienced during or in the month after pregnancy was 2.5 (range: 0–14).

When broken down by the four stress categories, the most common reported life stress was financial 65.7% (*n* = 224). Partner-associated stress followed at 49.6% (*n* = 169) with traumatic and emotional at 29.9% (*n* = 102) and 29.3% (*n* = 100), respectively.

As indicated on Table 2, among those initiating breastfeeding, across the four stress categories breastfeeding cessation rates were similar; within each stress type, a larger proportion of mothers experiencing stress stopped any and exclusive breastfeeding compared with mothers who did not report that type of stress. For example, the proportion of mothers who experienced financial stress who had stopped any BF by 4 weeks was 25.9% compared to 13.9% among mothers who did not report financial stress. Comparable numbers were found in the two of the other categories (partner associated: 25.5% versus 19.7%; traumatic: 27.2% versus 16.3%) but not for emotional where cessation was higher among those not reporting this type of stress (20.0% versus 22.5%). For the three other breastfeeding cessation outcomes, mothers experiencing all stress types were more likely to have stopped. Cessation of any breastfeeding by 13 weeks among mothers who experienced stress ranged across stress categories from 63.3% to 48.3%. For cessation of exclusive breastfeeding by 4 weeks among mothers reporting stress, the proportions ranged from 74.5% to 60.9% and for those reporting cessation by 13 weeks the range was 93.1% to 76.2%.

As shown in Table 3, crude analyses comparing the 4 different life stress categories and the 4 breastfeeding cessation outcomes result in odds ratios (OR) > 1.0 in all but 1 of the 16 comparisons; experiencing emotional stress during or in the month after pregnancy was not a risk factor for cessation of any breastfeeding by 4 weeks. Five relationships were statistically significant: financial stress and duration at 4 weeks (OR = 2.26; 95% CI: 1.16, 4.40); traumatic stress and exclusivity at both 4 and 13 weeks (OR = 1.86; 95% CI: 1.07, 3.25; OR = 3.94; 95% CI: 1.61, 9.61, resp.), and at 13 weeks partner-associated stress with both duration and exclusivity (OR = 1.82; 95% CI: 1.15, 2.88; OR = 2.80; 95% CI: 1.46, 5.37, resp.). Consistent with the findings in Table 2, all significant relationships indicated that in each category of life stress those experiencing the stress were more likely to stop breastfeeding. 

As shown in Table 4, we conducted 16 different logistic regression models to analyze the relationship between each of the 4 stress categories and the 4 breastfeeding cessation outcomes while adjusting for maternal factors identified a priori. After these adjustments, significant associations remained for only two relationships. 

Among low income mothers who initiated breastfeeding, those experiencing financial stress were nearly three times more likely to have stopped any breastfeeding at 4 weeks (OR = 2.76; 95% CI: 1.25, 6.06). In addition those who planned to breastfeed longer and were high school educated were also at higher risk to stop any breastfeeding by 4 weeks (OR = 2.89; 95% CI: 1.52, 5.50; OR = 2.54; 95% CI: 1.14, 5.64, resp.). Financial stress was not significantly associated with any of the other breastfeeding outcomes although BF plan remained significant as it was in all of the 16 models. 

The second model that showed a significant relationship to breastfeeding outcomes was traumatic stress. It was associated with cessation of exclusive breastfeeding by 13 weeks (OR = 2.95; 95% CI: 1.04, 8.38). Mothers with longer breastfeeding plans were also at higher risk for cessation (OR = 12.50; 95% CI: 4.03, 38.77), although this should be interpreted with caution given the wide confidence interval. Traumatic stress was not significantly associated with any of the other breastfeeding outcomes. 

While controlling for stress, several maternal factors were found to *decrease* the likelihood of early cessation. These included depression and cessation by 13 weeks in all four of the different stress type models (financial: OR = 0.38; 95% CI: 0.19, 0.75; emotional: OR = 0.45; 95% CI: 0.23, 0.88; traumatic: OR = 0.38; 95% CI: 0.19, 0.76; partner associated: OR = 0.46; 95% CI: 0.24, 0.92). Current alcohol use and/or smoking use were also protective factors for cessation of any breastfeeding by 13 weeks in all but the financial stress model (emotional: alcohol use: OR = 0.66; 95% CI: 0.46, 0.95; smoking: OR = 0.37; 95% CI: 0.17, 0.76; traumatic: smoking: OR = 0.37; 95% CI: 0.18, 0.77; partner associated: smoking: OR = 0.37; 95% CI: 0.18, 0.77). 

For the exclusivity models, while stress was not significant at 4 weeks cessation, higher BMI (>30.0) was also protective in all but financial stress (emotional: OR = 0.37; 95% CI: 0.17, 0.77; traumatic: OR = 0.40; 95% CI: 0.19, 0.83; partner associated: OR = 0.38; 95% CI: 0.18, 0.80). Similarly being of Black race also reduced the likelihood of cessation of exclusive breastfeeding by 13 weeks in both the financial and emotional stress models (OR = 0.21; 95% CI: 0.08, 0.57; OR = 0.23; 95% CI: 0.09, 0.61). 

## 4. Discussion

Among a convenience sample of over 340 low-income, primarily urban-dwelling mothers who initiated breastfeeding, not surprisingly over half reported financial stress and nearly half reported partner-associated stress. Across all four types, stress was associated with earlier cessation of any and exclusive breastfeeding. Crude relationships in three of the four stress categories had at least one statistically significant association with breastfeeding cessation. Once adjusted for other maternal factors (e.g., breastfeeding plan, depression, smoking, and alcohol use), the only significant relationships with breastfeeding outcomes were for mothers reporting financial or traumatic stress. Impact of stress on breastfeeding may differ by type of stress.

Despite methodological differences, our findings are consistent with the one breastfeeding and stress analysis by Li et al. (2008). [29]. Their study was from a different country (Australia), and the authors' operationalized stress differently and focused on a heterogeneous rather than only a low-income population. Despite these differences, they also demonstrated a significant relationship between stressful life events and the likelihood of early cessation of breastfeeding.

Other significant relationships in all four models were found with the outcome of cessation of any breastfeeding at 13 weeks. Stressed mothers who were depressed, smoked, and, for emotional stress, used alcohol were less likely to stop breastfeeding. These findings were inconsistent with other studies [17, 20]. Interestingly depression, alcohol use and smoking were not significant factors in early cessation of any breastfeeding (i.e., 4 weeks), which is consistent with the literature [16, 18, 19]. Mothers may receive breastfeeding support in the early postpartum period that dampens the negative influence of depression, alcohol use, and smoking on breastfeeding. Additionally, alcohol and cigarette use may be mechanisms used by mothers to self-medicate to help manage stress or depression [33, 34] and hence their protective association with breastfeeding outcomes. 

The relationship between stressful life events and depression is likely complex [33]. While it seems reasonable that mothers experiencing stress would stop breastfeeding earlier, other findings are less intuitive. Mothers reporting depression during pregnancy were less likely to stop any breastfeeding by 13 weeks across all stress categories. However, this finding is consistent with research showing that breastfeeding may buffer negative mood [35] and decrease stress hormone levels [36]. Another study found breastfeeding mothers to be more relaxed compared to bottle feeding mothers [37]. Further it is possible that for low income mothers the association previously identified between depression and breastfeeding outcomes is a proxy for the impact of stress [19]. Finally, while some literature suggests an indirect relationship between postpartum depression and breastfeeding outcomes, further interpretation of this significant relationship in our study is limited as the item used asked about depression during or right after the pregnancy?” (yes/no) and not postpartum depression per se [38, 39].

Plans for longer breastfeeding were significantly and independently associated with earlier cessation of any and exclusive breastfeeding. This differs from other studies where having a specific goal for breastfeeding (e.g., 6 months), considered a proxy for maternal self-efficacy, was associated with delayed cessation of breastfeeding [15, 18, 21]. Stress may mitigate against the mother's ability to achieve her plan. Specific to our findings, a mother planning to breastfeed longer, if she has financial stress may not be able to maintain breastfeeding to achieve her goal. Our findings reaffirm the broad impact that stress may have on breastfeeding self-efficacy.

Black mothers are commonly found to have a lower breastfeeding and exclusivity rates [11, 12]. While race was included in all 16 models, it was only statistically significant in one (emotional stress and exclusivity at 13 weeks) and it was protective. Other factors associated with race may be stronger predictors, or Black race, as it is used in other analyses, may not be a factor by itself but serve as a proxy for other barriers to sustaining breastfeeding in that subpopulation.

### 4.1. Limitations

The sample used for this analysis was drawn from a convenience sample of low-income mothers. Due to the relatively small sample, we may not have adequate power to detect statistically significant relationships for some outcomes. It is possible that some of the significant relationships were spurious findings particularly if a factor was a significant predictor at 4 but not at 13 weeks or vice versa. As this was an exploratory analysis, the stress categories did not adjust for the extent to which the respondent experienced physiologic stress (e.g., elevated cortisol levels). Data used in these analyses were self-report and are subject to the biases associated with these types of data. Breastfeeding outcomes assessed within 3 years are considered reliable, and our data collection was well within that timeframe [40]. Additionally our analyses do not account for other factors that may be associated with breastfeeding such as interpersonal support or hospital factors. 

## 5. Conclusions

Not all stressful life events may have an impact on cessation of any or exclusive breastfeeding among low-income women. Impact may differ by stress type. Stress may interfere with the achievement of the mother's breastfeeding goal. The association of depression, smoking, and alcohol use on breastfeeding cessation after adjusted for stress may indicate that breastfeeding serves as a coping mechanism. More research is needed to understand the relationship between breastfeeding plan, stress, other maternal factors, and their impact on breastfeeding outcomes. 

## Figures and Tables

**Table 1:** Maternal characteristics.

	*n* (%) *n* = 341
Age	
*n*, Mean	325, 26.3
[Minimum–Maximum]	[14.0–45.0]
Race^a^	
White	87 (26.1)
Black	132 (39.6)
Hispanic	84 (25.2)
Mixed and other	30 (9.0)
Language spoken at home^a^	
English only	238 (70.6)
English and Spanish	56 (16.6)
Spanish or other only	22 (6.5)
Other bi-multi-lingual	21 (6.2)
Education^a^	
<High school	56 (16.4)
≥High school	254 (74.5)
Unknown	31 (9.1)
Inner-City residence^b^	
Yes	220 (64.5)
BF Plan^a^	
Less than 3 months/none	149 (44.1)
4+ Months	189 (55.9)
Parity and BF	
First baby	157 (46.0)
Breastfed prior Infant—Yes	148 (43.4)
Breastfed prior Infant—No	36 (10.6)
Self-Reported depression during or right after most recent pregnancy	
Yes	90 (26.4)
Prepregnancy BMI^a^	
<25.0	152 (46.6)
25–29.9	87 (26.7)
>30.0	87 (26.7)
Alcohol use^a^	
Never/previous	170 (50.1)
Current use	169 (49.9)
Smoking^a^	
Not now/never	251 (74.9)
Current use	84 (25.1)
Overall stress	
*n*, Mean	325, 2.5
[Minimum–Maximum]	[0.0–11.0]
Financial stress^c^	
Yes	224 (65.7)
Emotional stress^d^	
Yes	100 (29.3)
Traumatic stress^e^	
Yes	102 (29.9)
Partner-Associated stress^f^	
Yes	169 (49.6)

Abbreviations: BF: breastfeeding; BMI: Body mass index.

^a^ *n* < 341.

^b^Defined using 9 urban zip codes with elevated levels of poverty.

^c^Financial stress defined as answering yes to one of the following occurring during or in the month after pregnancy: big problems with money, moved to a new address.

^d^Emotional stress defined as answering yes to one of the following occurring during or in the month after pregnancy: family member had serious accident/illness, husband/partner had serious accident/illness, husband/partner died, friend/relative died.

^e^Traumatic stress defined as answering yes to one of the following occurring during or in the month after pregnancy: stayed in a shelter or needed emergency shelter, physical fights with partner/husband or someone else, husband/partner had legal trouble and/or went to jail, mother had legal trouble and/or went to jail, someone very close had a problem with drinking or drugs.

^f^Partner-Associated stress defined as answering yes to one of the following occurring during or in the month after pregnancy: separated from husband/partner, argued with husband/partner more often, husband/partner said he did not want pregnancy.

**Table 2:** Breastfeeding cessation outcomes across maternal stress categories (*n* = 341).

	Categories of stress experienced							
	Financial^a^		Emotional^b^		Traumatic^c^		Partner-Associated^d^	
	Yes	No	Yes	No	Yes	No	Yes	No
	*n* = 224	*n* = 115	*n* = 100	*n* = 231	*n* = 102	*n* = 233	*n* = 169	*n* = 172
	*n* (%)	*n* (%)	*n* (%)	*n* (%)	*n* (%)	*n* (%)	*n* (%)	*n* (%)
Cessation of any BF								
by 4 Weeks	58 (25.9)	16 (13.9)	20 (20.0)	52 (22.5)	26 (25.5)	46 (19.7)	46 (27.2)	28 (16.3)
by 13 Weeks	126 (56.3)	64 (55.7)	60 (60.0)	125 (54.1)	61 (59.8)	125 (53.6)	107 (63.3)	83 (48.3)
Cessation of exclusive BF^e^								
by 4 Weeks	157 (70.1)	70 (60.9)	70 (70.0)	151 (65.4)	76 (74.5)	148 (63.5)	121 (71.6)	107 (62.2)
by 13 Weeks	191 (85.3)	90 (78.3)	83 (83.0)	190 (82.3)	95 (93.1)	182 (78.1)	151 (89.3)	131 (76.2)

Abbreviations: BF: breastfeeding.

^a^Financial stress defined as answering yes to one of the following occurring during or in the month after pregnancy: big problems with money, moved to a new address. (missing data from 2 mothers).

^b^Emotional stress defined as answering yes to one of the following occurring during or in the month after pregnancy: family member had serious accident/illness, husband/partner had serious accident/illness, husband/partner died, friend/relative died (missing data from 10 mothers).

^c^Traumatic stress defined as answering yes to one of the following occurring during or in the month after pregnancy: stayed in a shelter or needed emergency shelter, physical fights with partner/husband or someone else, husband/partner had legal trouble and/or went to jail, mother had legal trouble and/or went to jail, someone very close had a problem with drinking or drugs (missing data from 6 mothers).

^d^Partner-Associated stress defined as answering yes to one of the following occurring during or in the month after pregnancy: separated from husband/partner, argued with husband/partner more often, husband/partner said he did not want pregnancy (missing data from 0 mothers).

^e^Breastfeeding exclusivity was defined as the infant receiving only breast milk and no other liquid or solid food. This calculated outcome was based on mothers self-report of length of breastfeeding (infant age when last received breastmilk) and age when the infant first received formula, other liquids cereal, or solid foods.

**Table 3:** Comparison of stress categories and breastfeeding outcomes modeling for breastfeeding cessation.

	Cessation of any BF by 4 weeks		Cessation of any BF by 13 weeks		Cessation of exclusive^e^ BF by 4 weeks		Cessation of exclusive^e^ BF by 13 weeks	
	OR (95% CI)	*P* value	OR (95% CI)	*P* value	OR (95% CI)	*P* value	OR (95% CI)	*P* value
Financial stress^a^		**0.02**		0.68		0.13		0.17
Yes	2.26 (1.16, 4.40)		1.11 (0.68, 1.80)		1.47 (0.89, 2.43)		1.55 (0.32, 2.89)	
Emotional stress^b^		0.34		0.30		0.32		0.79
Yes	0.73 (0.38, 1.39)		1.31 (1.79, 2.18)		1.32 (0.77, 2.28)		1.10 (0.56, 2.15)	
Traumatic stress^c^		0.29		0.16		**0.03**		**<0.01**
Yes	1.38 (0.76, 2.48)		1.43 (0.86, 2.36)		1.86 (1.07, 3.25)		3.94 (1.61, 9.61)	
Partner-Associated stress^d^		0.06		**0.01**		0.10		**<0.01**
Yes	1.73 (0.98, 3.04)		1.82 (1.15, 2.88)		1.50 (0.92, 2.42)		2.80 (1.46, 5.37)	

Note: Reference is “no” for all 4 types of stress. Modeling for breastfeeding cessation. OR score 1< indicates more likely to breastfeed; OR score 1> indicates less likely to breastfeed.

Abbreviations: BF: breastfeeding.

^a^Financial stress (*n* = 339) defined as answering yes to one of the following occurring during or in the month after pregnancy: big problems with money, moved to a new address.

^b^Emotional stress (*n* = 331) defined as answering yes to one of the following occurring during or in the month after pregnancy: family member had serious accident/illness, husband/partner had serious accident/illness, husband/partner died, friend/relative died.

^c^Traumatic stress (*n* = 335) defined as answering yes to one of the following occurring during or in the month after pregnancy: stayed in a shelter or needed emergency shelter, physical fights with partner/husband or someone else, husband/partner had legal trouble and/or went to jail, mother had legal trouble and/or went to jail, someone very close had a problem with drinking or drugs.

^d^Partner-Associated stress (*n* = 341) defined as answering yes to one of the following occurring during or in the month after pregnancy: separated from husband/partner, argued with husband/partner more often, husband/partner said he did not want pregnancy.

^e^Breastfeeding exclusivity was defined as the infant receiving only breast milk and no other liquid or solid food. This calculated outcome was based on mothers self-report of length of breastfeeding (infant age when last received breastmilk) and age when the infant first received formula, other liquids cereal, or solid foods.

**Table 4:** Stress logistic regression for breastfeeding duration and exclusivity outcomes modeling for breastfeeding cessation.

	Cessation of any BF by 4 Weeks		Cessation of any BF by 13 Weeks		Cessation of Exclusive^g^ BF by 4 Weeks		Cessation of Exclusive^g^ BF by 13 Weeks	
	OR (95% CI)	*P*-value^a^	OR (95% CI)	*P*-value^a^	OR (95% CI)	*P*-value^a^	OR (95% CI)	*P*-value^a^
Financial Stress^b^ *n* = 298 [Reference]								
Financial Stress [No]		**0.02**		0.29		0.18		0.80
Yes	2.76 (1.25, 6.06)		0.70 (0.36, 1.35)		1.51 (0.82, 2.78)		1.11 (0.51, 2.42)	
Race/Ethnicity [White]		NS		NS		NS		**0.02**
Black	—		—		—		0.21 (0.08, 0.57)	
Hispanic	—		—		—		0.41 (0.12, 1.41)	
Mixed/Other	—		—		—		0.33 (0.08, 1.39)	
Education [<High School]		**0.04**		NS		NS		NS
Unknown	4.21 (0.85, 20.83)		—		—		—	
≥High School	2.54 (1.14, 5.64)		—		—		—	
Breastfeeding Plan [None/Unknown/Up to 3 months]		**<0.01**		**<0.01**		**<0.01**		**<0.01**
4+ Months	2.89 (1.52, 5.50)		6.80 (3.71, 12.46)		4.94 (2.65, 9.24)		10.91 (3.54, 33.66)	
Depression^c^ [No]		NS		**<0.01**		NS		NS
Yes	—		0.38 (0.19, 0.75)		—		—	
BMI [<25.0]		NS		NS		**0.01**		NS
25.0–29.0	—		—		1.13 (0.58, 2.18)		—	
>30.0	—		—		0.36 (0.17, 0.77)		—	

Emotional Stress^d^ *n* = 291 [Reference]								
Emotional Stress [No]		0.11		0.91		0.53		0.79
Yes	0.56 (0.27, 1.14)		1.04 (0.54, 1.99)		1.23 (0.65, 2.33)		0.89 (0.38, 2.09)	
Race/Ethnicity [White]		NS		NS		NS		**0.22**
Black	—		—		—		0.23 (0.09, 0.61)	
Hispanic	—		—		—		0.41 (0.12, 1.45)	
Mixed/Other	—		—		—		0.26 (0.06, 1.22)	
Breastfeeding Plan [None/Unknown/Up to 3 months]		**<0.01**		**<0.01**		**<0.01**		**<0.01**
4+ Months	2.82 (1.48, 5.39)		6.65 (3.60, 12.29)		4.56 (2.44, 8.52)		10.60 (3.45, 32.63)	
Depression^c^ [No]		NS		**0.02**		NS		NS
Yes	—		0.45 (0.23–0.88)		—			
BMI [<25.0]		NS		NS		**0.02**		NS
25.0–29.0	—		—		1.03 (0.52, 2.01)		—	
>30.0	—		—		0.37 (0.17, 0.77)		—	
Alcohol Use [Never/Not Now]		NS		**0.03**		NS		NS
Current Use	—		0.66 (0.46, 0.95)		—		—	
Smoking [Never/Not Now]		NS		**<0.01**		NS		NS
Current Use	—		0.37 (0.17, 0.76)		—		—	

Traumatic Stress^e^ *n* = 295[Reference]								
Traumatic Stress [No]		0.71		0.61		0.17		**0.04**
Yes	1.14 (0.57, 2.27)		0.84 (0.43, 1.64)		1.59 (0.82, 3.12)		2.95 (1.04, 8.38)	
Mom Age		NS		**0.04**		NS		NS
Continuous	—		1.07 (1.00, 1.13)		—			
Breastfeeding Plan [None/Unknown/Up to 3 months]		**<0.01**		**<0.01**		**<0.01**		**<0.01**
4+ Months	2.66 (1.41, 5.01)		7.24 (3.92, 13.39)		5.24 (2.78, 9.85)		12.50 (4.03, 38.77)	
Depression^c^ [No]		NS		**<0.01**		NS		NS
Yes	—		0.38 (0.19, 0.76)		—		—	
BMI [<25.0]		NS		NS		**0.02**		NS
25.0–29.0	—		—		1.11 (0.57, 2.17)		—	
>30.0	—		—		0.40 (0.19, 0.83)		—	
Smoking [Never/Not Now]		NS		**<0.01**		NS		NS
Current Use	—		0.37 (0.18, 0.77)		—		—	

Partner-Associated Stress^f^ *n* = 300[Reference]								
Partner-Associated Stress [No]		0.11		0.30		0.79		0.11
Yes	1.71 (0.88, 3.30)		1.37 (0.76, 2.48)		1.08 (0.61, 1.93)		1.92 (0.86, 4.31)	
Education [<High School]		**<0.05**		NS		NS		NS
Unknown	3.89 (0.77, 19.63)		—		—		—	
≥High School	2.53 (1.14, 5.60)		—		—		—	
Breastfeeding Plan [None/Unknown/Up to 3 months]		**<0.01**		**<0.01**		**<0.01**		**<0.01**
4+ Months	2.84 (1.51, 5.37)		7.39 (4.01, 13.63)		4.84 (2.61, 9.00)		11.46 (3.72, 35.36)	
Depression^c^ [No]		NS		**0.03**		**<0.05**		NS
Yes	—		0.46 (0.24, 0.92)		0.50 (0.25, 0.99)		—	
BMI [<25.0]		NS		NS		**0.02**		NS
25.0–29.0	—		—		1.09 (0.57, 2.11)		—	
>30.0	—		—		0.38 (0.18, 0.80)		—	
Smoking [Never/Not Now]		NS		**<0.01**		NS		NS
Current Use	—		0.37 (0.18, 0.77)		—		—	

Note: Reference is “no” for all 4 types of stress. Modeling for breastfeeding cessation. OR score 1< indicates more likely to breastfeed; OR score 1> indicates less likely to breastfeed.

Variables Included in Model: age (continuous), race, language, education, inner-city residence, breastfeeding plan, parity and breastfeeding, depression, BMI, alcohol use, smoking.

Abbreviations: BF: Breastfeeding; NS: not significant.

^a^Global *P*-value.

^b^Financial stress defined as answering yes to one of the following occurring during or in the month after pregnancy: big problems with money, moved to a new address.

^c^Self-reported depression during or right after pregnancy.

^d^Emotional stress defined as answering yes to one of the following occurring during or in the month after pregnancy: family member had serious accident/illness, husband/partner had serious accident/illness, husband/partner died, friend/relative died.

^e^Traumatic stress defined as answering yes to one of the following occurring during or in the month after pregnancy: stayed in a shelter or needed emergency shelter, physical fights with partner/husband or someone else, husband/partner had legal trouble and/or went to jail, mother had legal trouble and/or went to jail, someone very close had a problem with drinking or drugs.

^f^Partner-Associated stress defined as answering yes to one of the following occurring during or in the month after pregnancy: separated from husband/partner, argued with husband/partner more often, husband/partner said he did not want pregnancy.

^g^Breastfeeding exclusivity was defined as the infant receiving only breast milk and no other liquid or solid food. This calculated outcome was based on mothers self-report of length of breastfeeding (infant age when last received breastmilk) and age when the infant first received formula, other liquids cereal, or solid foods.
